# Vortex trapping recaptures energy in flying fruit flies

**DOI:** 10.1038/s41598-021-86359-z

**Published:** 2021-03-26

**Authors:** Fritz-Olaf Lehmann, Hao Wang, Thomas Engels

**Affiliations:** 1 Department of Animal Physiology, University of Rostock, Albert-Einstein-Str. 3, 18059 Rostock, Germany; 2 Jiangsu Provincial Key Laboratory of Bionic Functional Materials, College of Mechanical and Electrical Engineering, Nanjing University of Aeronautics and Astronautics, 29 Yudao St., Nanjing, 210016 China

**Keywords:** Physiology, Zoology

## Abstract

Flapping flight is one of the most costly forms of locomotion in animals. To limit energetic expenditures, flying insects thus developed multiple strategies. An effective mechanism to reduce flight power expenditures is the harvesting of kinetic energy from motion of the surrounding air. We here show an unusual mechanism of energy harvesting in an insect that recaptures the rotational energy of air vortices. The mechanism requires pronounced chordwise wing bending during which the wing surface momentary traps the vortex and transfers its kinetic energy to the wing within less than a millisecond. Numerical and robotic controls show that the decrease in vortex strength is minimal without the nearby wing surface. The measured energy recycling might slightly reduce the power requirements needed for body weight support in flight, lowering the flight costs in animals flying at elevated power demands. An increase in flight efficiency improves flight during aversive manoeuvring in response to predation and long-distance migration, and thus factors that determine the worldwide abundance and distribution of insect populations.

## Introduction

To allocate low energy investment for big gains is an universal rule in nature^1–8^. The reduction of energy spent during locomotion is thus key to the ecological success of many flying animals. During wing flapping of insects, the energy for propulsion stems from the contraction of flight muscles. The majority of muscle energy during flight appears as kinetic energy in the surrounding air but part of it is also stored as elastic potential energy in the muscle tissue^9^, as elastic deformations of the thorax^10^ and wings^11^, and as kinetic energy in the movement of wings. Energy loss in deforming structures are common in flight. Fly wings, for example, elastically recycle only ~ 80–90% of their deformation energy during wing flapping^11,12^, making flight less efficient. Many animals thus benefit from a reduction of energy needed to accelerate the surrounding fluid by extracting kinetic energy from their environment. Birds in flocks, for example, take advantage of flight formations using vortices shed from the wing tips of the heading animals^13–16^ and trouts save muscle power by exploiting rotational energy from vortices generated by bodies upstream^17^.

Vortices at the wing may enhance the production of locomotor forces in flight. Elevated leading edge suction in birds^18,19^, bats^20,21^ and insects^4,5,22,23^ is generated by the wing’s leading edge vortex (LEV) that boosts lift production for weight support and increases locomotor capacity during flight manoeuvres. The cyclic changes in wing flapping direction within the stroke cycle, however, force insects to periodically shed these vortices into the wake. Thus, at each stroke reversal, a large amount the kinetic energy and thus muscle power is wasted, lowering propulsion efficiency owing to energy conservation laws^4^. To cope with these constraints, many insects developed strategies to simultaneously increase lift production and locomotor efficiency during wing flapping. Dragonfly hindwings, for example, utilize energy-rich vortex flows shed from the forewings that increases aerodynamic efficiency^24^ and butterfly wings lower the kinetic energy of stopping vortices by slicing through it^5^. While physical and numerical modelling of flight in small insects showed that a single wing recaptures vortex flows from the wake of a previous halfstroke^4,22,25,26^, two-winged models apparently miss this ability^27–30^.

**Figure 1:**
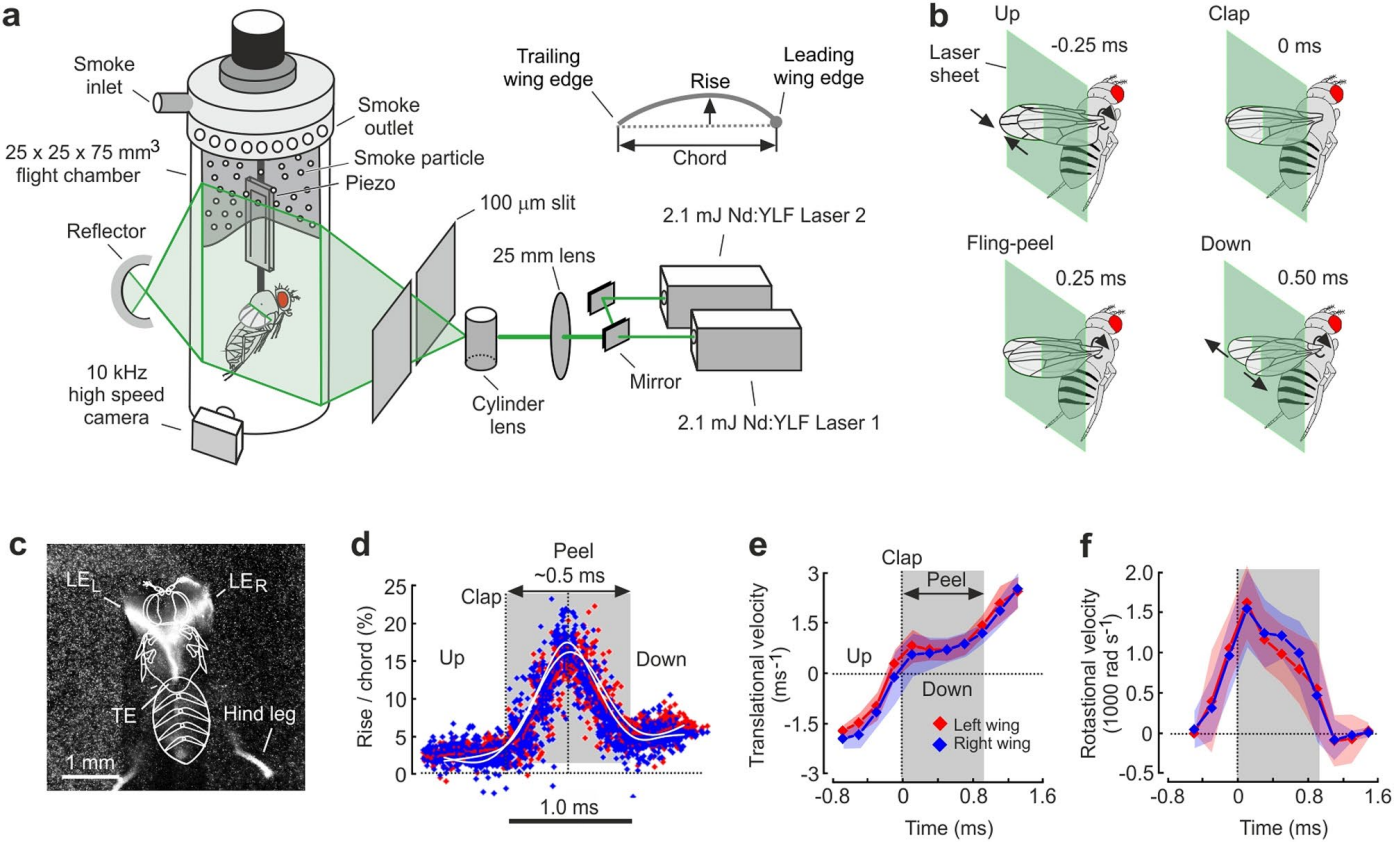
Experimental setup, wing kinematics and elastic wing deformation during the dorsal stroke reversal. **(a**) A single fly is tethered to a 100 µm tungsten wire and flown in a 25 × 25 × 75 mm^3^ flight chamber. The wire is attached to a piezo element recording an electrical signal synchronous to wing flapping motion. The signal triggers a high-speed video camera equipped with magnifying lenses and a teleconverter. A Nd:YLF dual laser generates a ~ 500 µm thin light sheet for flow measurements by time-resolved particle image velocimetry. Flow vectors are calculated from adaptive cross-correlation and smoothed by a spatial filter with 160 × 160 image pixels. The laser light is guided through a 25 mm beam waist adjuster and a cylinder lens, and narrowed by a ~ 100 µm mechanical slit. Smoke from burning plant debris is pumped as seeding particles into the chamber at low speed. Inset shows schematics of the wing’s rise-to-chord ratio as plotted in d. Drawing is not to scale. (**b**) Four significant phases of wing motion during dorsal wing-wing interaction in the fruit fly. At the stroke reversal, leading edges of left and right wing touch (up) while the wings rotate about their longitudinal axes. After wing clapping (clap), the wings chordwise bend (peel), forming an opening cleft (fling) at the leading edge while the trailing edges remain connected. The wings separate ~ 500 µs after wing clapping, starting the downstroke (down). (**c**) Top view image of left and right wing chord of *Drosophila virilis* during the fling phase, highlighted by a laser at 0.6 wing length. Body outline is superimposed. View is on the dorsal body side and from wing tip to hinge. (**d**) Change in chordwise wing bending (rise-to-chord). Left (red) and right (blue) wings are maximally deformed ~ 300 µs after wing clapping. White lines are filtered means. (**e**) Mean translational and (**f**) mean rotational wing velocity about the wing’s longitudinal axis measured at 0.6 wing length of left (red) and right (blue) wing during the dorsal reversal. Corresponding coloured areas indicate standard deviations. Data are from 12 animals and 15 stroke cycles. Grey areas show wing peel time.

This study identifies that the 1.3 mg fruit fly *Drosophila virilis* harvests energy from trapped vortices during clap-and-fling wing kinematics^27,30–34^, at which the flapping wings physically touch and chordwise bend during the dorsal stroke reversal (Fig. 1). Experiments previously showed that clap-and-fling kinematics alters vortex development^35,36^ and also lowers the energetic costs for lift production per unit flight muscle mass compared to flight without clap-and-fling kinematics^29,37,38^. In freely flying fruit flies, high-speed video recording shows that full and partial dorsal wing-wing contact occurs in ~ 33% of 144 tested wing strokes (N = 24 flies, supplementary text S1), suggesting that this kinematic manoeuvre is frequent in fruit flies. Figure 1b shows wing orientation in 4 out of 17 measured times during the dorsal rotation phase of a tethered fruit fly. The images suggests that clap-and-fling wing motion in fruit flies lasts approximately one millisecond or ~ 15% of the ~ 150 Hz stroke cycle. We estimated the wing’s chordwise deformation using high-speed videography and a laser sheet (Fig. 1a,c), illuminating wing profiles at 4 equally distant wing sections. Figure 1d shows the evolution of the chordwise deformation at the centre of aerodynamic force production at ~ 0.6 wing length^29^.

During wing rotation at the dorsal reversal, the wings deform only little until they clap together (0 ms stroke cycle, Fig. 1b). However, they strongly deform within only ~ 0.5 ms during wing separation (peel motion), increasing the wings’ rise-to-chord ratio up to ~ 18% (Fig. 1d). The flexing motion of the wing chord accordingly reduces the elevated drag on the wing that results from both the viscous forces between the wing surfaces and wing acceleration during wing separation^30,31^. Figure 1d shows that wing rotation occurs symmetrical in fruit flies with respect to the up- and downstroke, producing elevated lift for weight support compared to a delayed rotation^4^. At wing clap, instantaneous translational wing velocity is minimum but quickly accelerates to ~ 2.4 ms^−1^ within only ~ 1.5 ms during wing separation (Fig. 1e). The wings’ rotational motion peaks at ~ 1579 rad s^−1^ during wing clapping and decelerates to zero at the beginning of the downstroke within less than a millisecond (Fig. 1f). In contrast to the negative wing camber as used in computer simulations^30,39^, the wings in fruit flies have a small positive camber at the beginning of the downstroke. This positive camber contributes to lift production at the initial phase of the downstroke^40^ because it increases the wing’s angle of attack and thus fluid momentum^41^.

**Figure 2:**
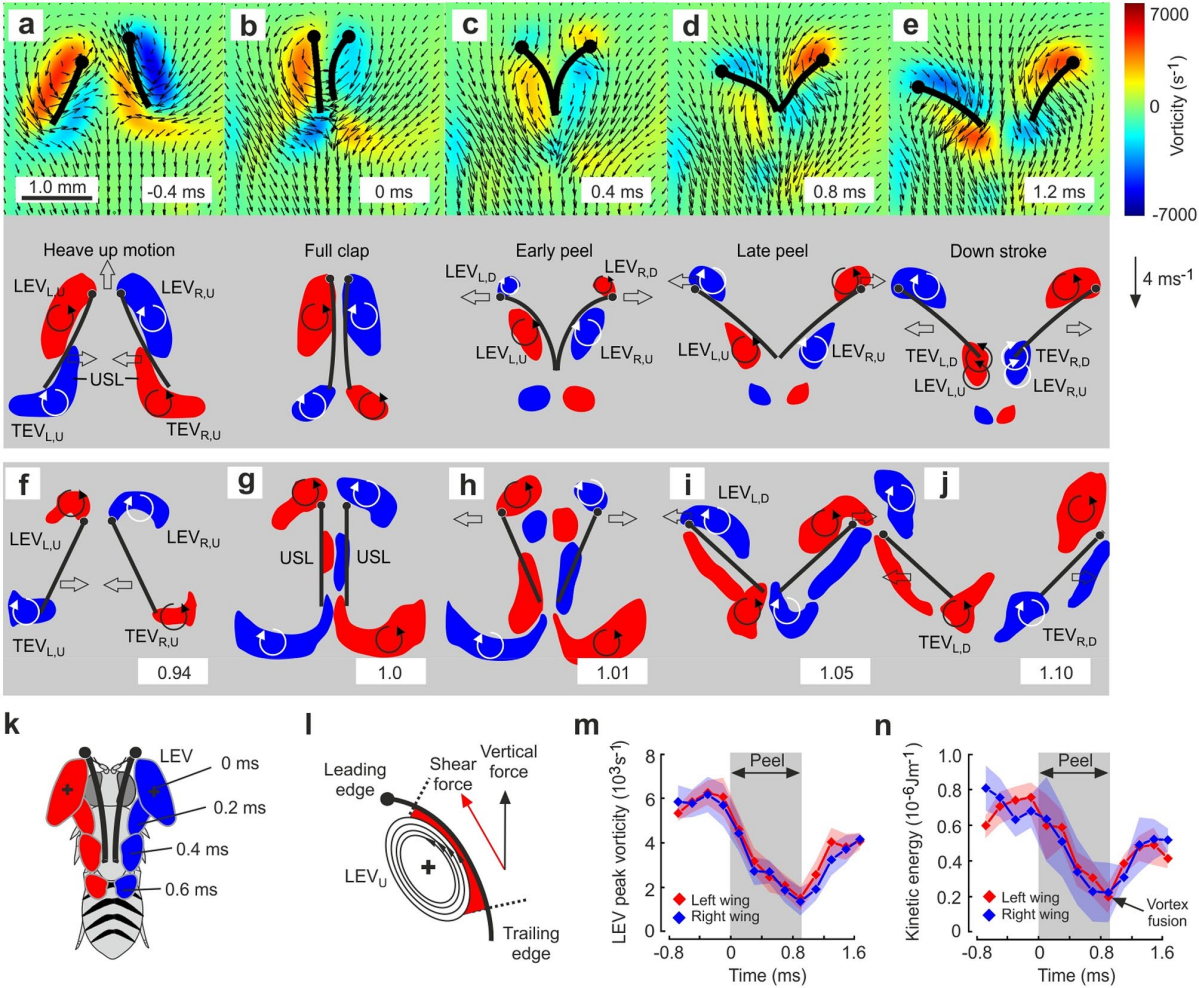
Fluid vorticity, LEV travelling and depletion of vorticity and kinetic energy. **(a–e**) Images show fluid vorticity (upper row) and vortex reconstruction (lower row) at 5 out of 17 measured times (10 kHz sampling rate) during clap-peel-fling in a single wing stroke cycle. Black lines highlight chordwise deformation of left and right wing blade at 0.6 wing length with the wing’s leading edges (solid circle) pointing upward. *LEV* leading edge vortex; *TEV* trailing edge vortex; *USL* undershear layer; *L* left wing; *R* right wing; *U* wing upstroke; *D* wing downstroke. Clockwise and anti-clockwise circulation is shown in blue and red, respectively. Vortices from the upstroke are completely shed during the dorsal stroke reversal. At the beginning of the downstroke, the leading edge vortex from the upstroke fuses with the trailing edge vortex that forms at the beginning of the downstroke. Vortices in the lower row were reconstructed from 15 stroke cycles and wing sections at 0.6 wing length. (**f–j**) Vortex reconstruction at 0.65 wing length in rigid robotic wings mimicking clap-and-fling in fruit fly wing kinematics. Timing is shown in fraction of the stroke cycle (1). Flapping frequency was ~ 0.17 Hz and Re =  ~ 180. Data are replotted from a previous study^28^. (**k**) Reconstruction of leading edge vortex shape and chordwise travelling during dorsal stroke reversal after clapping (0 ms). (**l**) Sketch of hypothesized momentum transfer of rotational energy (enstrophy) to the close-by surface by friction between leading edge vortex and wing surface. The horizontal wing stroke plane in fruit flies during hovering flight favours frictional forces in a vertical direction, supporting lift and nose-down pitching moments. (**m–n**) Depletion of peak vorticity and chordwise sectional energy of the leading edge vortex within ~ 1.0 ms after wing clapping (0 ms). LEV from the upstroke is shed after wing clapping and regains strength by fusion with the trailing edge vortex ~ 0.8 ms after the stroke reversal. Vortex decay time of a shed trailing edge vortex is shown in supplementary text S2. Data are means from 90 stroke cycles recorded in 6 animals and shaded areas indicate standard deviation.

The fate of vortical structures at the end of the wings’ upstroke is key for aerodynamic recapture mechanisms (movie S1). Figure 2 shows the evolution of leading and trailing edge vortices during the dorsal stroke reversal in 5 equally-spaced out of 17 moments sampled at 10 kHz. At the beginning of wing rotation, vorticity of LEV at the ventral wing surface increases due to the wing’s increasing angle of attack. As a consequence of wing rotation and wing deceleration, the wing also produces counter-balancing vorticity at the trailing edge (Fig. 2a). At wing clapping, both vortices have gained maximum strength but remain attached at the ventral wing surface (Fig. 2b). The stretched shape of LEV and trailing edge vortices results from air viscosity at low Reynolds number of ~ 214 for wing flapping in conjunction with the vortex shear velocity relative to the air. Throughout wing peel motion (Fig. 2c), the LEV does not separate from the wing as reported for robotic wings^28^ and computer simulation models^29,30^. Instead, it remains close to the wing’s ventral surface and consequentially travels downstream from the leading towards the trailing wing edge (Fig. 2d). The ability of fruit fly wings to trap the LEV from the upstroke results from the combined effect of chordwise wing bending during peel and upward heaving motion^27^. The two wing surfaces effectively separate left and right LEV, avoiding that both vortices cancel each other out owing to their opposite spins. This factor saves the LEV’s kinetic energy for later use. The positive wing camber generates a curvature with finite radius, which increases the tangential contact area between wing and LEV and inevitably facilitates vortex trapping. For comparison, in rigid wings of robotic *Drosophila* wing flapping models, leading edge vorticies are shed during the fling phase into the opening cleft between both wings^28^ (Fig. 2f–j) that forces vortex extinction according the Kelvin’s law rather than energy recycling.

The time, at which the moving upstroke LEV reaches the wing’s trailing edge (~ 1 ms after wing clap, Fig. 2d,e), the wings start to separate and to produce start vortices that balance the wings’ bound circulation. At this moment, upstroke LEV and downstroke start vortex fuse because of equal spin, and are subsequently shed as a single structure into the wake (Fig. 2e). The latter process is quantified in Fig. 2m, showing that the LEV gains ~ 2.9 times its vorticity (~ 1442 ± 568 s^−1^ to ~ 4112 ± 314 s^−1^) during fusion. While travelling chordwise, the trapped LEV keeps its orientation relative to ventral wing surface (Fig. 2k). This potentially allows to transfer the LEV’s kinetic energy to the wing by surface friction (Fig. 2l). Due to the horizontal stroke plane in flying fruit flies, the resulting shear force vector at the wing may contribute to both vertical lift support and nose down pitching moments on the fly body for posture control. Notably, Fig. 2m shows that while travelling, the LEV looses ~ 74.2% or ~ 4356 ± 738 s^−1^ of its initial peak vorticity (~ 5873 ± 909 s^−1^, N = 15 stroke cycles, 12 animals) within less than a millisecond. The LEV’s sectional kinetic energy decreases likewise by ~ 71% from ~ 718 ± 93 nJm^−1^ before peel to ~ 212 ± 126 nJm^−1^ after peel (Fig. 2n). At absence of steering manoeuvres elicited by visual stimulation of the fly’s compound eyes, the difference in vortex decay is negligible between both wings.

The assumption that vortex energy is transferred to the wing is supported by comparison of slopes for vorticity decay time between leading and trailing edge vortices (TEV) in the fly (supplementary text S2). These data show that vortex circulation decreases ~ 6.4 to ~ 9.0 times and sectional energy ~ 6.1 to ~ 7.8 times faster in the LEV than the TEV (supplemental text S3). We further confirmed this finding by comparing the time evolution of vorticity in vortices modelled by a Lamb-Oseen vortex model (supplementary text S3) and by estimation of vorticity decay times of free vortices shed from a robotic fruit fly wing (supplementary text S4 and S5). There is also no evidence for vortex-splitting at the wing surface that could cause vorticity decrement^42^. We also tested the idea that spanwise axial flow on the wing surface pulls the LEV outward, in turn changing both sectional vorticity and energy of the LEV over time (supplementary text S4). Due the LEV’s conical vorticity profile, a change in LEV’s longitudinal position relative to the wing surface might appear as a change in vorticity in the 2-dimensional measurement plane during particle image velocimetry. Data measured in a fruit fly model wing, however, suggest that spanwise displacement of a vortex may account for not more than ~ 21.9% change in circulation and ~ 36.6% change in sectional kinetic energy (supplementary text S4). These findings rule out the hypothesis that spanwise drift causes the measured attenuation in LEV strength in the fruit fly during peel motion. We also carefully considered vortex stretching as a possible explanation for vortex decay using computational fluid dynamics (supplementary text S6). Vortex stretching is a pure 3-dimensional effect and cannot be captured considering our 2-dimensional slices of the flow field. Both, enstrophy dissipation and vortex stretching determine the time evolution of enstrophy. The numerical simulations show that vortex stretching is several orders of magnitude smaller than enstrophy dissipation (supplementary text S6). The excessive dissipation observed in leading edge vortices near the wings can thus not be explained by vortex stretching and supports the hypothesis that energy is transferred to the wing. In addition, negligible vortex stretching also implies that 2-dimensional measurements are sufficient to characterize the decay of vortex enstrophy. Altogether, the controls suggest that the LEV’s loss of kinetic energy during peel motion cannot be fully explained by enstrophy dissipation, vortex stretching or axial displacement.

To answer the question on how much muscle mechanical power a fruit fly might maximally save by vortex trapping, we determined total energy loss in both the fly’s LEV and a corresponding Lamb-Oseen vortex model, included the LEV’s spanwise conical vorticity profile and calculated the transferred power with respect to the flapping cycle (supplementary text S3). We found that the LEV might maximally transfer ~ 1.08 nJ kinetic energy to the wing surface. The potential kinetic energy transfer converts to ~ 0.66 Wkg^-1^ flight muscle mass and further to ~ 1.1% the total aerodynamic power requirements for flight (~ 59.8 W kg^−1^ flight muscle mass)^43^ or ~ 3.0% the total induced power requirements (~ 21.4 W kg^−1^ flight muscle mass)^43^ for vertical lift production^44^ during hovering flight. However, if we compare with mean induced power during peel time (supplementary text S3), LEV trapping might lower induced power during peel by ~ 20.6%. Moreover, in a horizontal stroke plane, the transferred energy likely accelerates the wings upward, which might further augment lift production due to augmentation of the transient unilateral asymmetry in vorticity distribution during fling motion^30^. As the leading edge vortices merge with trailing edge vortices at the end of wing separation, the proximity of LEVs to the wing surfaces does not hinder the generation of bound circulation during wing translation. Thus, despite vortex trapping, Weis-Fogh’s original clap-and-fling mechanism^32^ still applies, as two strong new downstroke LEVs are generated at the leading edge during fling motion (Fig. 2e). The latter enhance the wing’s total circulation and thus lift production during the early phase of the downstroke.

Worldwide, small insects are of significant ecological^45,46^ and agricultural^47^ importance. Savings in muscle power by recycling of kinetic energy from the wake extends the locomotor capacity of these animals. An increase in locomotor capacity is highly relevant for the abundance of insect populations and their migration^7,8^ but also in behavioural contexts, in which an insect requires elevated aerodynamic forces such as during take-off^48^, fast forward flight^49^, climbing flight^50^, evasive manoeuvring during predation^6^, and chasing flight^51–53^. Assuming that insect flight is primarily limited by the production of muscle power and not the production of aerodynamic forces, any increase in locomotor efficiency should provide a benefit for a species flying at elevated power demands^54^. This conclusion is evident considering the low total flight efficiency in insects, ranging from ~ 2.3% to ~ 3.5%^55^. Our study, moreover, provides an explanation on how flies utilize elastic deformation of their wings for energy harvesting and thus sheds light on the general function of wing elasticity in flapping flight of insects. The latter aspect is currently under extended controversial debate, not only for wing flapping motion in animals^56–60^ but also for bioinspired man-made flight devices^61–63^.

## Data availability

All data is available in the main text or the supplementary materials.

## Supplementary Information

Supplementary Video.

Supplementary Information.

## References

[1] Alexander RM (2005). Models and the scaling of energy costs for locomotion. J. Exp. Biol..

[2] Marden JH (2000). Variability in the size, composition, and function of insect flight muscles. Annu. Rev. Physiol..

[3] Ellington CP (1999). The novel aerodynamics of insect flight: applications to micro-air vehicles. J. Exp. Biol..

[4] Dickinson MH, Lehmann F-O, Sane S (1999). Wing rotation and the aerodynamic basis of insect flight. Science.

[5] Srygley RB, Thomas ALR (2002). Unconventional lift-generating mechanisms in free-flying butterflies. Nature.

[6] Combes SA, Rundle DE, Iwasaki JM, Crall JD (2012). Linking biomechanics and ecology through predator-prey interactions: flight performance of dragonflies and their prey. J. Exp. Biol..

[7] Wainwright CE, Reynolds DR, Reynolds AM (2020). Linking small-scale flight manoeuvers and density profiles to the vertical movement of insects in the nocturnal stable boundary layer. Sci. Rep..

[8] Krauel JJ, Brown VA, Westbrook JK, McCracken GF (2018). Predator–prey interaction reveals local effects of high-altitude insect migration. Oecologia.

[9] Dickinson MH (2005). Molecular dynamics of cyclically contracting insect flight muscle in vivo. Nature.

[10] Lau GK, Chin YW, Goh JT-W, Wood RJ (2014). Dipteran-insect-inspired thoracic mechanism with nonlinear stiffness to save inertial power of flapping-wing flight. IEEE Trans. Robot..

[11] Lehmann, F.-O., Gorb, S., Nasir, N. & Schützner, P. Elastic deformation and energy loss of flapping fly wings. *J. Exp. Biol.* **l214**, 2949–2961 (2011). 10.1242/jeb.045351 21832138

[12] Wehmann, H.-N., Heepe, L., Gorb, S. N., Engels, T. & Lehmann, F.-O. Local deformation and stiffness distribution in fly wings. *Biol. Open* **8**, bio038299 (2019). 10.1242/bio.038299 PMC6361194 30642916

[13] Weimerskirch H, Martin J, Clerquin Y, Alexandre P, Jiraskova S (2001). Energy saving in flight formation. Nature.

[14] May RM (1979). Flight formations in geese and other birds. Nature.

[15] Bajec IL, Heppner FH (2009). Organized flight in birds. Anim. Behav..

[16] Biewener AA (2018). Animal locomotion: near-ground low-cost flights. Curr. Biol..

[17] Liao JC, Beal DN, Lauder G (2003). Fish exploiting vortices decrease muscle acitivity. Science.

[18] Videler JJ, Stamhuis EJ, Povel GDE (2004). Leading-edge vortex lifts swifts. Science.

[19] Warrick DR, Tobalske BW, Powers DR (2009). Lift production in the hovering hummingbird. Proc. R. Soc. Lond. B.

[20] Hedenström A (2007). Bat flight generates complex aerodynamic tracks. Science.

[21] Muijres FT (2008). Leading-edge vortex improves lift in slow-flying bats. Science.

[22] Birch JM, Dickinson MH (2001). Spanwise flow and the attachment of the leading-edge vortex on insect wings. Nature.

[23] Ellington, C. P., Berg, C. v. d., Willmott, A. P. & .Thomas, A. L. R. Leading-edge vortices in insect flight. *Nature* **384**, 626–630 (1996).

[24] Usherwood JR, Lehmann F-O (2008). Phasing of dragonfly wings can improve aerodynamic efficiency by removing swirl. J. R. Soc. Interface.

[25] Wang ZJ, Birch JM, Dickinson MH (2004). Unsteady forces and flows in low Reynolds number hovering flight: two-dimensional computations *vs* robotic wing experiments. J. Exp. Biol..

[26] Birch JM, Dickinson MH (2003). The influence of wing-wake interactions on the production of aerodynamic forces in flapping flight. J. Exp. Biol..

[27] Lehmann F-O, Pick S (2007). The aerodynamic benefit of wing–wing interaction depends on stroke trajectory in flapping insect wings. J. Exp. Biol..

[28] Lehmann FO, Sane SP, Dickinson MH (2005). The aerodynamic effects of wing-wing interaction in flapping insect wings. J. Exp. Biol..

[29] Miller LA, Peskin CS (2005). A computational fluid dynamics of 'clap and fling' in the smallest insects. J. Exp. Biol..

[30] Miller LA, Peskin CS (2009). Flexible clap and fling in tiny insect flight. J. Exp. Biol..

[31] Cheng X, Sun M (2017). Aerodynamic forces and flows of the full and partial clap-fling motions in insects. PeerJ.

[32] Weis-Fogh T (1973). Quick estimates of flight fitness in hovering animals, including novel mechanisms for lift production. J. Exp. Biol..

[33] Arora N, Gupta A, Sanghi S, Aono H, Shyy W (2014). Lift-drag and flow structures associated with the “clap and fling” motion. Phys. Fluids.

[34] Jones, S. K., Laurenza, R., Hedrick, T. L., Griffith, B. E. & Miller, L. A. Lift vs. drag based mechanisms for vertical force production in the smallest flying insects. *J. Theor. Biol.* **7**, 105–120, 10.1016/j.jtbi.2015.07.035 (2015). 10.1016/j.jtbi.2015.07.035 26300066

[35] Maxworthy, T. Experiments on the Weis-Fogh mechanism of lift generation by insects in hovering flight Part 1. Dynamics of the 'fling'. *J. Fluid Mech.* **93**, 47–63 (1979).

[36] Kolomenskiy D, Moffatt H, Farge M, Schneider K (2011). Two-and three-dimensional numerical simulations of the clap–fling–sweep of hovering insects. J. Fluid Struct..

[37] Marden JH (1987). Maximum lift production during takeoff in flying animals. J. Exp. Biol..

[38] Sun M, Yu X (2006). Aerodynamic force generation in hovering flight in a tiny insect. AIAA J..

[39] Vanella M, Fitzgerald T, Preidikman S, Balaras E, Balachandran B (2009). Influence of flexibility on the aerodynamic performance of a hovering wing. J. Exp. Biol..

[40] Zhao L, Huang Q, Deng X, Sane SP (2010). Aerodynamic effects of flexibility in flapping wings. J. R. Soc. Interface.

[41] Engels T, Wehmann H-N, Lehmann F-O (2020). Three-dimensional wing structure attenuates aerodynamic efficiency in flapping fly wings. J. R. Soc. Interface.

[42] Boghosian M, Cassel K (2016). On the origins of vortex shedding in two-dimensional incompressible flows. Theor. Comp. Fluid Dyn..

[43] Lehmann F-O, Dickinson MH (1997). The changes in power requirements and muscle efficiency during elevated force production in the fruit fly, *Drosophila melanogaster*. J. Exp. Biol..

[44] Ellington, C. P. The aerodynamics of hovering insect flight. VI. Lift and power requirements. *Phil. Trans. R. Soc. Lond. B* **305**, 145–181 (1984).

[45] Austin, A. & Dowton, M. *Hymenoptera: Evolution, Biodiversity and Biological Control*. (Csiro Publishing, 2000).

[46] Crespi, B. J., Carmean, A., David, A. & Chapman, T. W. Ecology and evolution of galling thrips and their allies. *Annu. Rev. Entomol.* **42**, 51–71 (1997). 10.1146/annurev.ento.42.1.51 15012307

[47] Terry I (2001). Thrips and weevils as dual, specialist pollinators of the Australian cycad *Macrozamia communis* (Zamiaceae). Int. J. Plant Sci..

[48] Sunada S, Kawachi K, Watanabe I, Azuma A (1993). Performance of a butterfly in take-off flight. J. Exp. Biol..

[49] Ellington, C. P. The aerodynamics of hovering insect flight. III. Kinematics. *Proc. R. Soc. Lond. B* **305**, 41–78 (1984).

[50] Cooter RJ, Baker PS (1977). Weis-Fogh clap and fling mechanism in *Locusta*. Nature.

[51] Land MF, Collett TS (1974). Chasing behaviour of houseflies (*Fannia canicularis*). J. Comp. Physiol. A.

[52] Wehrhahn, C., Poggio, T. & Bülthoff, H. Tracking and chasing in house flies (*Musca*). An analysis of 3-D flight trajectories. *Biol. Cybern.* **45**, 123–130 (1982).

[53] Wehrhan C (1979). Sex-specific differences in the chasing behavior of houseflies (*Musca*). Biol. Cybern..

[54] Ellington CP (1991). Limitations on animal flight performance. J. Exp. Biol..

[55] Lehmann F-O (2001). The efficiency of aerodynamic force production in *Drosophila*. Comp. Biochem. Physiol. Part A.

[56] Du G, Sun M (2012). Aerodynamic effects of corrugation and deformation in flapping wings of hovering hoverflies. J. Theor. Biol..

[57] Moses KC, Michaels SC, Willis M, Quinn RD (2017). Artificial *Manduca sexta* forewings for flapping-wing micro aerial vehicles: how wing structure affects performance. Bioinsp. Biomim..

[58] Nakata T, Liu H (2012). A fluid-structure interaction model of insect flight with flexible wings. J. Comp. Phys..

[59] Mountcastle AM, Combes SA (2013). Wing flexibility enhances load-lifting capacity in bumblebees. Proc. R. Soc. Lond. A.

[60] Zheng L, Hedrick TL, Mittal R (2013). Time-varying wing-twist improves aerodynamic efficiency of forward flight in butterflies. PLoS ONE.

[61] Widhiarini S (2016). Bird-mimetic wing system of flapping-wing micro air vehicle with autonomous flight control capability. J. Bionic. Eng..

[62] Shyy W (2010). Recent progress in flapping wing aerodynamics and aeroelasticity. Prog. Aero. Sci..

[63] Yu Y, Guan Z (2015). Learning from bat: Aerodynamics of actively morphing wing. Theor. Appl. Mech. Lett..

