# Supplementary material for: Vortex trapping recaptures energy in flying fruit flies
Source: Sci Rep. 2021 Mar 26;11:6992. doi: 10.1038/s41598-021-86359-z (PMC7997922; doi:10.1038/s41598-021-86359-z)
Supplement: Supplementary file 2 — Supplementary Information. [file 41598_2021_86359_MOESM2_ESM.pdf]

Supplementary Materials for

Vortex trapping recaptures energy in flying fruit flies

Fritz-Olaf Lehmann, Hao Wang and Thomas Engels

Correspondence to: fritz.lehmann@uni-rostock.de

This PDF file includes:

Materials and Methods
Supplementary Text
Figs. S1 to S11
Caption for Movie S1

Other Supplementary Materials for this manuscript include the following:

Movie S1

Materials and Methods

Animals, tethering procedure, and wing kinematics. We tested 15 female, 3-5 days old fruit flies *Drosophila virilis* from an inbred lab strain. Body mass, wing length and mean wing chord of these animals were ~1.64±0.23 mg, ~2.79±0.16 mm, ~1.03±0.06 mm (means ±S.D.), respectively. For flight, the animals were anesthetized at 4°C on a cold plate and tethered to a 100 µm tungsten wire using blue-light activated glue. Mean stroke period after tethering was ~6.67±0.14 ms (N=51 wing beats, 6 flies). As tethering conditions typically augment wing stroke amplitudes in fruit flies, the tethered animals yielded full physical wing contact at the clap phase during 57±7% (mean ±S.D.) of all stroke cycles (90 cycles, 6 animals). Control measurements show that in freely flying fruit fly females, physical wing contact during the dorsal stroke reversal occurs in ~4.1% (full wing clap)[1] and ~33% (partial wing clap)[2] of 144 stroke cycles (N=24 flies, supplementary text S1). Reynolds number for wing flapping in *Drosophila virilis* based on mean wing tip velocity (u_{tip} = 2.34 ms^{-1}) and mean wing chord (c = 1.03 mm) is ~160.

Experimental setup. The tethered flies were flown in a 25 x 25 mm^2 wide, and 75 mm high glass chamber. The inside air was seeded with submicrometre-sized particles of olive seed smoke, generated by a custom designed smoke generator. The smoke was humidified, filtered, and electrically pumped into the flight chamber at low speed. Controls showed that the smoke had no toxic effect on the animals. A commercial digital Particle-Image Velocimetry (DPIV) system (DynamicStudio, version: 2.30.47.0) was employed for flow visualization. The system consisted of a double-pulsed laser (Litron LDY-304, 2.1 mJ per laser pulse, dual head: DualPower 20-1000) and a synchronizer running at high repetition rate. The laser was directed through a 100 µm narrow mechanical slit, and a cylinder optics with 25mm focal length produced a sheet of ~500µm thickness inside the flight chamber. Small slit size and minimum laser power avoided substantial heat effects on the wing, preventing changes in the wing's sensory feedback and biomechanical properties[3]. A coated glass reflector returned the laser light that passed through the chamber towards the animal, in order to illuminate the wings from both sides. The laser sheet intersected the wing either at ~0.2, ~0.4, ~0.6, and ~0.8 wing length during bending measurements or at ~0.6 wing length during flow measurements. Images were taken by a charge-coupled device high-speed camera (Phantom V12.1), equipped with an 36mm extension ring, a macro lenses (AF Macro Nikkor, 60mm), a 2-fold teleconverter (Sigma EX DG), a magnifying lens (Raynox DCR-250), and appropriate spectral filters. The camera was synchronized with the laser and captured image pairs (512 x 536 pixels) at 10 KHz frame rate and with temporal spacing of Δt = 25-50 µs.

Digital particle image velocimetry analysis. Air velocity fields for each interrogation window within the recorded images were calculated from adaptive cross-correlation. In each tested fly, we sampled 17 images with 100 µs temporal spacing for the complete clap-and-fling motion at dorsal stroke reversal. Cross-correlation started with areas of 128 x 128 images pixels and was refined to 32 x 32 pixels with 50% overlap. The algorithm was a high sub-pixel accuracy algorithm and mean displacement was ~8 pixels or ~1/4 of the interrogation window size. Measurement uncertainty was not more than ±1%. Vorticity and flow vectors were smoothed by a spatial

averaging filter with 160 x 160 image pixels. Velocity vectors that stem from the moving wing section (~10 vectors per image) were replaced by interpolation of neighbouring fluid vectors. Before averaging, data were phase-corrected in time to consider differences in frequency between various stroke cycles and various animals. A self-developed image-processing tool written in MatLab® was used to identify vortices inside PIV images (Fig. S2, supplementary text S2). Owing to the vortices' flat shape and fusing boundaries, we determined vortex location using a threshold value (isoline) of 80% maximum core vorticity. As a measure for wing blade-sectional kinetic energy, we calculated local kinetic energy from the product of fluid mass and the magnitude of translational velocity within the vortex area of the uppermost 20% vortex vorticity values (Fig. 2, supplementary text S3).

Total sectional kinetic energy and power of leading and trailing edge vortices were estimated from a numerical viscous Lamb-Oseen vortex model fitted to the measured 20% uppermost vorticity values (supplementary text S3). Vorticity evolution of an ideal vortex without both nearby surfaces and neighbouring vortices was determined by developing the Lamb-Oseen vortex in time. To estimate the changes in vorticity of the conical leading edge vortex in the wing's span-wise direction, we used measurements obtained in a dynamically-scaled robotic wing of a fruit fly, flapping at similar Reynolds number and kinematics (160 deg stroke amplitude, 0.26 Hz stroke frequency, 45° angle of attack at midstroke, ±10% symmetrical timing of wing rotation at the stroke reversals, triangular velocity profiles, horizontal stroke plane; supplementary text S4). Robotic experiments were also used to experimentally confirm the measured vorticity decay times of the fly's leading and trailing edge vortices and to approximate axial flow velocity from the wing hinge to wing tip at the stroke reversals (supplementary text S4 and S5). Data are presented as means ± standard deviation.

Supplementary text

Supplementary text S1: Three-dimensional reconstruction of wing kinematics in free flight

To score clap-and-fling wing kinematics in freely flying fruit flies, we have flown *Drosophila melanogaster* females in a cylindrical free flight arena that had 70 mm diameter and 62 mm height. The arena was equipped with an back-illuminated visual random dot pattern for visual guidance. The animals voluntarily started from a platform inside the arena. Body and wing motion of the subsequent flight manoeuvres were 3-dimensionally tracked at 3500 frames s^{-1} using three high-speed video cameras (Fastcam-X 1024 PCI, Photron). At ~200Hz wing stroke frequency, temporal resolution was ~17.5 frames per stroke cycle. For tracking, body and wings are marked with fluorescent dot markers (200 µm diameter, 0.1 µg weight). Three markers labelled the thorax and one marker each wing tip, leading wing edge at half wing length, and trailing edge. The cameras were equipped with appropriate optical filters and synchronized with 48 UV-light emitting diodes with a flash duration of ~60 µs to avoid motion blur. Recorded images were enhanced by image processing software and the markers' 3-dimensional position calculated using self-written software in MatLab®. From these values, we reconstructed body and wing motion in all 6 degrees-of-freedom throughout the stroke cycle[4].

Figure S1 shows that the animals use complete wing-clapping, i.e. when the distance between both wing tips is below 0.1mm, in 4.1% of the recorded stroke cycles. Partial wing clapping at which the wings have physical contact during peel at least at the trailing wing edges, frequently occurs in free flight in ~33% of all recorded wing strokes. Since the animals were preselected and starved for at least ~6h prior testing, their initial body mass (1.31 ±0.07 mg, *N*=61 flies) was slightly reduced by ~0.14 mg (~11% of initial mass) compared to non-starved animals. Thus, non-starved animals might show clap-and-fling kinematics more frequent, assuming that force enhancement by clap-and-fling is of functional relevance during flight in fruit flies[2]. The free flight analysis suggests that fruit flies regularly use clap-and-fling during manoeuvring flight, which is similar to many other insect species exhibiting this specific type of wing kinematics. It has also been shown that insects with clap-and-fling kinematics produce more lift per unit flight muscle mass than insects using conventional wing kinematics[5,6].

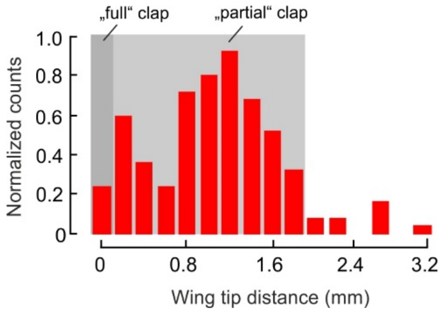

Fig. S1 | Wing tip distance at dorsal stroke reversal of freely flying fruit flies. Minimum distance between left and right wing tip during dorsal stroke reversal in 3- to 7 days old fruit flies *Drosophila melanogaster*. At full clap conditions the wings fully touch along the entire wing chord. At partial clap conditions, wings touch at least at their trailing edges during rotation. Data stem from *N*=24 flies and *n*=144 stroke cycles, and were 3-dimensionally reconstructed from high-video recordings during manoeuvring flight[4].

Supplementary text S2: Vortex reconstruction and time evolution

Leading edge vortices in fruit fly and robotic wings are typically flattened owing to low Reynolds number (Re~130) and their outer boundaries are often unclear. Vortices shed from leading and trailing edges may even fuse in the wake, which makes it difficult to estimate vortex shape and circulation, including the temporal decay of vorticity during wing flapping. Thus, to estimate total circulation of a vortex, we first identified vortices by an image processing tool, locating them according to their vorticity isoline at 56% (robotic wings) and 80% (fruit fly wings) peak (core) vorticity[7]. In an ideal vortex (Lamb-Oseen vortex, see below), the 56% and 80% vorticity isolines only cover ~44% and ~20% of total vortex circulation, respectively. Second, we assumed that circulation of a flattened vortex equals the circulation of an ideal round vortex. This ideal vortex, we numerically described using incompressible Navier-Stokes equations. The parameter of the equations were fitted to the experimental data, i.e. maximum circulation at the vortex core, the mean radius at 56% or 80% isoline, and the vorticity at the isolines. From the solution of the Navier-Stokes equations, we eventually estimated both the vortex's entire kinetic energy and its enstrophy.

The Navier-Stoke equations model a two-dimensional line vortex with a Gaussian vorticity profile in its cross-section[8]. These equations equal the Lamb-Oseen vortex model of an incompressible, viscous flow[9]. Lamb-Oseen models, for example, has been successfully validated in experiments with smoke rings at an intermediate Reynolds

number of 1850[10]. Mach number of *Drosophila virilis* wing motion is ~0.009, indicating incompressible flow conditions. Time evolution of vorticity in a Lamb-Oseen vortex and in polar coordinates (r, θ) is given by,

$$\omega(r,\theta,t)=\frac{\Gamma_0}{4\pi\nu(t_0+t)}e^{-\frac{r^2}{4\nu(t+t_0)}}. \qquad \text{(eq. S1)}$$

with Γ_0 the initial circulation contained in the vortex, v the kinematic viscosity of the fluid, r the distance of the vorticity isoline to the vortex centre (vortex radius), and t the time. The constant t_0 is determined by the initial radius (r_0) of the vortex core, $t_0 = 0.25 r_0 v^{-1}$. Kinematic viscosity is 15.1 x 10^{-6} m^2 s^{-1} for air. The azimuthal component of the velocity $u(r, \theta)$ equals,

$$u(r,\theta,t)=\frac{\Gamma_0}{2\pi r}\left(1-e^{-\frac{r^2}{4\nu(t+t_0)}}\right), \qquad \text{(eq. S2)}$$

and the radial component is zero. The radial profiles of vorticity and azimuthal velocity of a Lamb-Oseen vortex show that with increasing time the vorticity of the core peak decreases while the vortex increases in width. To estimate total vorticity and velocity of the measured vortex, we thus determined the radial vorticity profile by systematically varying Γ_0 and t in equation 1, until the profile matched both the measured vortex core vorticity at r=0 and the 56% (robotic experiments) or 80% (fruit fly) vorticity values at the measured radius r. The Lamb-Oseen model predicts that the characteristic radius of a vortex r^* increases with time by $r^*=\sqrt{4\nu(t+t_0)}$.

To experimentally validate the Lamb-Oseen modelling, we calculated radial profiles of vorticity using the 80% maximum vorticity isoline in vortices of the fruit fly (Fig. S2a). We then predicted their total vorticity at a 50% isoline using Lamb-Oseen modelling and compared these data with vorticity of our PIV measurements inside a 50% isoline area (Fig. S2b). We performed this validation for both the upstroke LEV that quickly decays and the stop vortex that is shed from the trailing edge prior wing clapping. Figure S2b shows that predicted values (open circles) largely match the measured data (solid) in leading (LEV) and trailing (TEV) edge vortices of both wings. Mean difference between theoretical prediction and measurement is negligible, amounting to 0.40% (left LEV), 0.18% (left TEV), 0.50% (right LEV), and 0.27% (right TEV). We conclude that the simple, 2-dimensional Lamb-Oseen model is a suitable method to predict vortex strength and temporal development during clap and fling wing motion in flies.

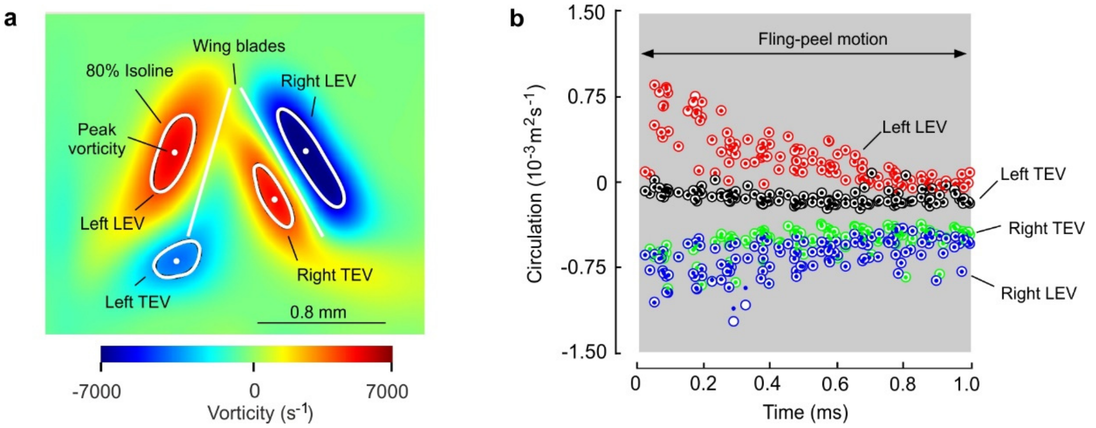

Fig. S2 | Reconstruction of total vorticity during clap-and-fling in *Drosophila*. a, Vorticity of left and right vortices prior dorsal wing clapping in tethered fruit flies. **b**, Validation of Lamb-Oseen vortex model on the radial profile for circulation during clap-and-fling wing motion in *Drosophila*. Dots indicate DPIV measurements of circulation of vortices within an area bounded by the vorticity isoline at 50% peak vorticity ($r_{50\%}$). Open circles shows circulation within a bounded area at $r_{50\%}$ of a Lamb-Oseen model at which the vorticity profile was reconstructed from the measured vorticity profile at 80% threshold ($r_{80\%}$). Figure shows data from a single animal and 10 stroke cycles (N=122 measurements of each vortex). LEV, leading edge vortex; TEV, trailing edge vortex partly superimposed by wing undershear layer.

Supplementary text S3: Vortex kinetic energy, enstrophy, and dissipation

Sectional kinetic energy of vortical fluid motion in a 2-dimensional Gaussian vortex per unit vortex length, E'_{SEC}, is defined as the integral over the entire plane of flow (x,y) as,

$$E'_{SEC}(t)=\frac{\rho}{2}\iint u^2(t)\,dx\,dy\,, \qquad \text{(eq. S3)}$$

with ρ=1.205 kg m^{-3} the density of air (fruit fly) and ρ=0.88·10^3 kg m^{-3} of paraffin (robotic model, see below), and u the magnitude of the measured velocity vector.

The theoretical energy dissipation rate of a free vortex (dE'_{SEC}/dt) may be approximated from enstrophy and kinematic viscosity. This relationship is,

$$\frac{dE'_{SEC}(t)}{dt} = -2\nu Z'_{SEC}(t) \qquad \text{(eq. S4)}$$

with Z the sectional, rotational energy (enstrophy) of a vortex,

$$Z'_{SEC}(t) = \frac{\rho}{2}\int\int \omega^2 dxdy \, . \qquad \text{(eq. S5)}$$

For a 2-dimensional Gaussian vortex, sectional enstrophy has a finite value and can be simplified to the equation,

$$Z'_{SEC}(t) = \frac{\rho\Gamma_0^2}{16\pi\upsilon(t+t_0)} \, . \qquad \text{(eq. S6)}$$

The latter estimate can be used for volumetric vortices whose length is larger then the core diameter. The appropriate constants for the fruit fly experiments are: (i) t=0 ms, (ii) an initial vortex size r_0=0.5c (see Materials and Methods section) with wing chord c=1.03 mm at 0.6 wing length that leads to the "vortex shape" parameter t_0=4.39 x 10^{-3} s (see supplemental text S2), and (iii) a left-right averaged initial circulation Γ_0=4.66 x 10^{-3} m^{2}s^{-1} of the LEVs measured at wing clap (t=0 ms, Fig. S3b). From these values we obtain a theoretical dissipation rate dE'_{SEC}/dt for a freely moving LEV of *Drosophila* of approximately –2.37 x 10^{-4} Js^{-1}m^{-1}.

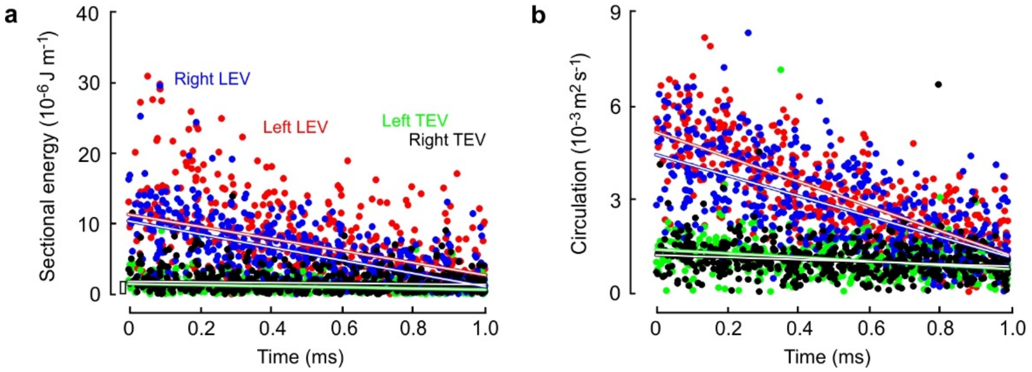

Fig. S3 | Sectional energy and total circulation of leading and trailing edge vortices during fling-peel motion in *Drosophila*. Total circulation was calculated from Lamb-Oseen models matched to vorticity profiles using isolines of 80% peak vorticity. **a**, Decrease of sectional kinetic energy of vortices at 0.6R from the wing base of left LEV (red), right LEV (blue), left TEV (green), and right TEV (black) during fling phase. Slopes of linear regression fit are -8.60 x 10^{-3}, -9.31 x 10^{-3}, -0.85 x 10^{-3}, and -0.44 x 10^{-3}, respectively. Regression offsets are 11.25 x 10^{-6}, 10.35 x 10^{-6}, 1.44 x 10^{-6}, and 1.70 x 10^{-6}, respectively. **b**, Slope of linear decrease in circulation is -3.92, -3.25, -0.61, and -0.36, respectively (linear regression fit). Initial circulation Γ_0 at wing clap is ~4.97 x 10^{-3} m^{2}s^{-1} for left LEV (red) and ~4.35 x 10^{-3} m^{2}s^{-1} for right LEV (blue). For colour coding see *a*. Data stem from 6 flies and 51 stroke cycles. N=122 measurements of each vortex.

While the above equations estimate energy of a sectional, 2-dimensional vortex, an estimation of a volumetric (2 components, 3-dimensional; 2C3D) vortex requires spatial spanwise integration of vorticity from wing base to tip in the flapping wing. These data were derived from previous measurements on relative vorticity of LEV of two robotic wings[2]. The robotic wings were upscaled models (~15 cm wing length) of a *Drosophila* wing and moved with a *Drosophila*-like kinematic pattern. Details of the experimental setup are previously described[11]. Circulation was measured at 6 wing blade sections 0.1R, 0.33R, 0.5R, 0.65R, 0.75R, and 0.95R and subsequently normalized to the measured LEV vorticity value at 0.6R (Γ*=1.0) of *Drosophila*. This was done by linearly extrapolating the data towards wing base and wing tip assuming zero circulation normal to the longitudinal wing axis at both positions and linearly interpolating the remaining values. The procedure generated normalized circulation of 0.75±0.20, 0.91±0.23, 0.89±0.22, 1.06±0.44, 0.90±0.45, and 0.38±0.35 (means±S.D.) at the 6 blade sections, respectively, and mean relative circulation from base to tip was Γ^*_{bt}=0.76±0.25 (mean±S.D., N=200 data).

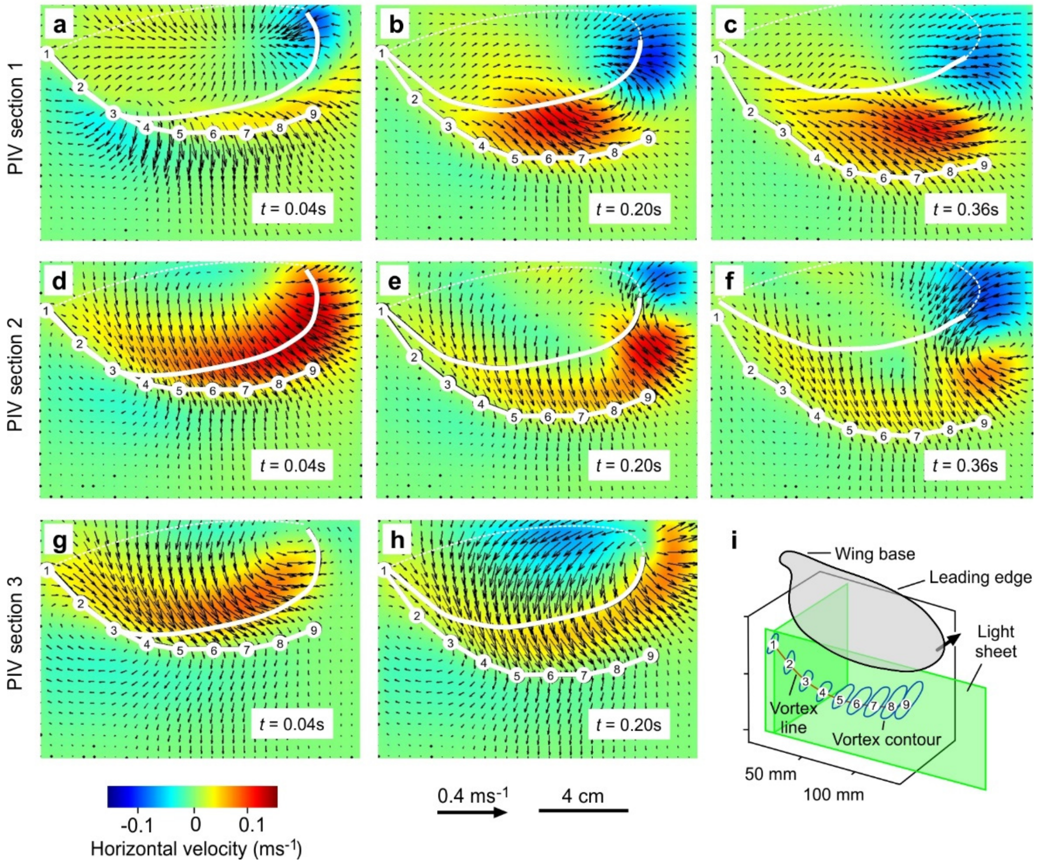

Fig. S4 | Wake structure behind the wing's trailing edge of a *Drosophila* model wing. Horizontal and vertical flow is shown for PIV planes orientated parallel to the wing surface at dorsal stroke reversal. **a-c**, PIV section 1 was nearest to the trailing wing; **d-f**, section 2 was at 20 mm distance; **g-h**, and section 3 at 40 mm distance behind the trailing edge at the transition from up- to down stroke. Data show times after stroke reversal (*t*= 0 ms, 90° wing angle of attack). **i**, Experimental setup. Wake structure was estimated in 3 planes orientated parallel to the wings surface at stroke reversal. Sectional vorticity of the start vortex (TEV) was measured in planes that were normally orientated to the wing's longitudinal axis at 9 equally spaced distances (1-9). Vortex contours show TEV isolines at 56% core vorticity. The wing's geometric angle of attack is ~4.4°, ~21.8°, and ~39.2° at 40ms, 200ms, and 360ms after the dorsal stroke reversal, respectively.

Using the latter value, we approximated the volumetric strength of the fly's LEV as shown in figure 2n. The approach ignores vortical components (swirl) in the spanwise direction. The LEV's total kinetic energy is derived from,

$$E_{LEV}(t) = 0.76RE'_{SEC}(t) \qquad \text{(eq. S7)}$$

with E'_{sec} the sectional energy measured for the wing blade at 0.6*R*.

As the integral over the plane of vortex flow in a 2-dimensional numerical Lamb-Oseen vortex is infinite, it contains infinite kinetic energy because velocity decays with r^{-1}. Total kinetic energy of a 2-dimensional vortex is thus constant and independent of time. The same holds for energy inside a core radius bounded by isolines at any threshold. To overcome this difficulty, we considered the vortex with finite radius, limiting vortex diameter to the generating and interacting structure, i.e. wing chord. The bounded domain for absolute kinetic energy estimates in *Drosophila virilis* we approximated by a circle with a radius of $c/2$ (r_0 = 0.515 mm). Considering altogether - the LEV's volume (2-components 3-dimensional) with vortex length equal to wing length, its conical shape in spanwise direction (factor 0.76, see above), and the measured loss in LEV sectional kinetic energy (Fig. 2n, ~0.51 x 10^{-6} J m^{-1}) after wing clapping (*t*=0), the total energy loss of a single upstroke LEV measured during t_{peel}=1.0 ms peel is equal to,

$$\Delta E_{LEV} = E_{LEV}(t) - E_{LEV}(t_{peel}), \qquad \text{(eq. S8)}$$

and amounts to ΔE_{LEV} = ~10.8·x 10^{-10} J. By contrast, the theoretical energy dissipation for a freely moving volumetric (2-component, 3-dimensional) vortex with an initial circulation Γ_0 = 4.66 x 10^{-3} m^2s^{-1} (mean of left and right wing, Fig. S3b), with mean dE'_{SEC}/dt=-2.37 x 10^{-4} $Js^{-1}m^{-1}$ (see above), t_{peel}=1.0 x 10^{-3} s, and wing length

R=2.79 x 10^{-3} m is:

$$\Delta E_{Diss} = R t_{peel} \left| dE'_{SEC} / dt \right|, \qquad \text{(eq. S9)}$$

and approximates ΔE_{Diss}=~6.61 x 10^{-10} J.

For comparison, the data in figure S3b suggest that LEV dissipates between 6.43 and 9.09-times faster than the shed TEV. The work by Clercx and Heijst[12] on dissipation of a 2-dimensional vortex in the presence of a solid wall even suggests higher values although under these conditions, energy cannot be turned in any movement but is fully converted into heat. At the lowest tested Reynolds number of ~600, their data show that dissipation of kinetic energy during vortex-wall interaction (periodic boundary conditions) is ~100-times higher than the dissipation of the travelling dipole due to diffusion (free vortex, no slip condition). Thus, the nearby wing surface in the fly might be more effective than our measurements and simulations currently suggest.

For further comparison, we determined the possible contribution of LEV kinetic energy to flight muscle mass-specific power requirements of the entire stroke cycle. Total energy E_{LEV} is converted to power P_{LEV} by the following equation,

$$P_{LEV} = \frac{2\Delta E_{LEV}}{t_{sc} m_{muscle}}, \qquad \text{(eq. S10)}$$

with m_{muscle} = 0.49 mg the flight muscle mass in *D. virilis*, i.e. ~30% of total body mass in fruit flies, stroke cycle period t_{sc}=~6.67ms, and the factor of 2 considers both wings[13]. LEV energy loss converts into P_{LEV}=~0.66 Wkg^{-1} flight muscle mass. This value compares to ~1.1% of total mechanical power requirements for hovering flight conditions in *Drosophila* (P_{mech}=59.8 Wkg^{-1}) and ~3.0% of induced power requirements (P_{ind}=21.4 Wkg^{-1}), i.e. the power needed to produce the vertical momentum supporting the animal's body weight[13]. If we roughly compare the contribution of energy transfer to cycle-averaged induced power during t_{peel}=1.0 ms (P^*_{ind}=21.4 Wkg^{-1} / 6.67), LEV capture lowers induced power requirements by 20.6%. In sum, although vortex trapping might deliver a short force pulse at the beginning of the downstroke, which may contribute to pitch control of the animal, it apparently saves only little of the total flight costs in the fruit fly. The latter, however, was not expected, considering the short period over which this mechanism works.

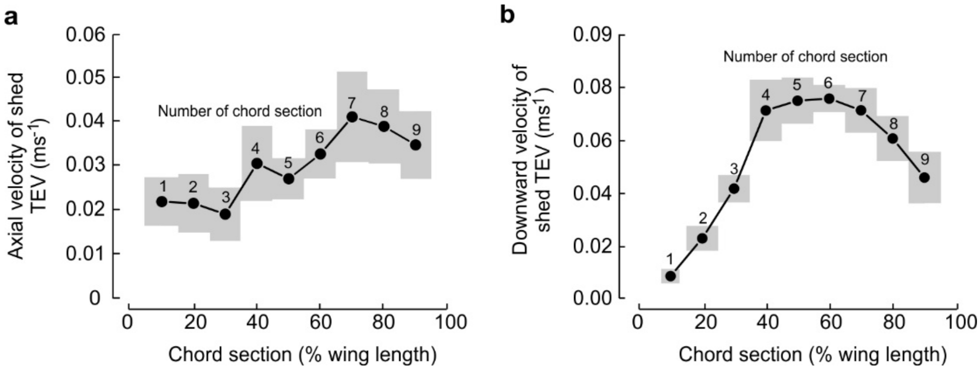

Fig. S5 | Wake velocity (axial flow) at the TEV vortex core of *Drosophila* model wings. a, Horizontal velocity; **b**, vertical wake velocity. Velocities were estimated at 9 sections of the TEV as shown in figure S4f. Means ±S.E.M. (grey area) were computed from 3 PIV planes (0mm, 20mm, 40mm behind leading wing edge, *cf.* figure 4f) and 3 times (t=40ms, t=200ms, and t=360 ms) after the dorsal stroke reversal. At 0.6R TEV axial velocity is 0.032 ms^{-1}. See figure S4 for numbering of chord sections.

Supplementary text S4: Robotic validation - axial flow estimation

Due to the LEV's conical shape with increasing diameter from wing base to tip[14,15], we tested the hypothesis that strong spanwise flow (axial flow) causes the decrease in the LEV's vorticity during fling-peel at 0.6R. There a contradicting measurements on axial flow in physical *Drosophila* models. One study[15] showed strong axial flow of the LEV core during mid downstroke and moderate axial flow on the remaining wing area. Another study reported negligible axial flow inside the LEV at Reynolds numbers typical for small insects[16]. In both measurements, however, the LEV was not free but attached to the wing surface and did not travel along the wing chord as shown in *Drosophila* (Fig. 2f-j). Thus, in the following experiment, we estimated axial flow in a TEV shed at the stroke reversals of a *Drosophila* model wing.

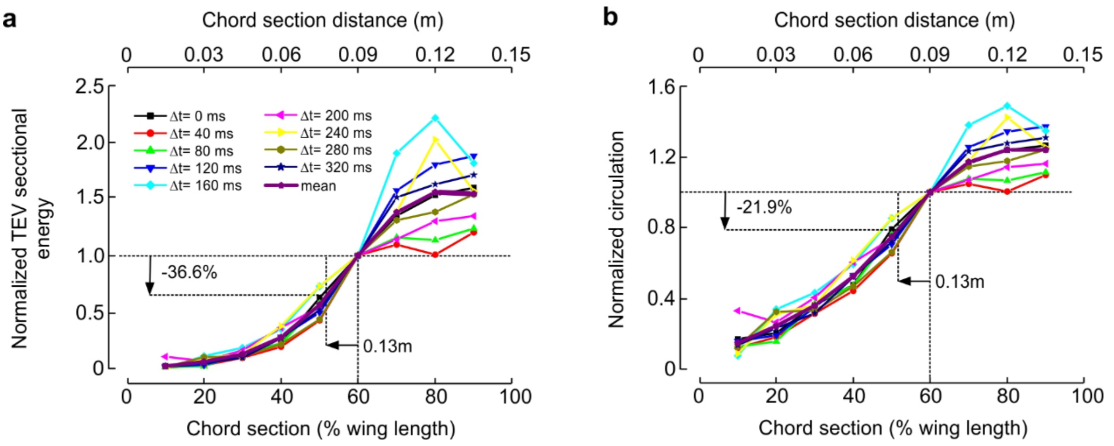

Fig. S6 | Spanwise vorticity profile of a shed trailing edge vortex (TEV) at the start of the downstroke of a *Drosophila* model wing. a, Sectional energy and **b**, sectional circulation were computed from isolines at 56% core vorticity of TEV and normalized to values measured at 0.6 wing length (*R*=15 cm). At a measured outbound axial flow velocity of ~0.032 ms^{-1} at 0.6*R*, the conical TEV vortex profile moves by ~0.13 m within 0.42s towards the wing tip. These changes might result in a ~36.6% decrease of sectional energy and ~21.9% sectional circulation.

We tested the hypothesis using a single model fruit fly wing (Plexiglas wing, *R*=150 mm, mean *c*=60 mm) and flapped it in a robotic apparatus with a generic kinematic pattern (160° horizontal flapping amplitude; 0.26 Hz flapping frequency; 45° morphological angle of attack at midstroke, ±10% symmetrical timing of wing rotation at the stroke reversals, triangular velocity profiles) in a horizontal plane. The wing flapped in a 0.43 m^3 tank filled with paraffin (Merkur CP 120; density, 0.88 x 10^3 kg m^{-3}; kinematic viscosity 1.2 x 10^{-4} m^2 s^{-1}) in order to match Reynolds number (~114) at the wing tip to the flying animal[12]. Flow measurements were performed using a 50 mJ per pulse dual mini-Nd:YAG laser (Insight v. 5.1, TSI Inc.) equipped with a 60mm lens to create two identical positioned light sheets approximately 5 mm thick separated in time by Δt = 5.0 ms. Paired images (~120mm x ~120mm) were captured using a PowerView 2M camera (TSI Inc., model 630157, 1200 x 1600 image pixels). A two-frame cross-correlation of pixel intensity using the Hart Correlator engine for a final interrogation area of 32 x 32 pixels, resulted in 38 x 50 vectors for an image pair. Data of each wing section stem from the 4th - 9th stroke cycle after onset of flapping motion to avoid initial transient effects.

We measured flow velocity and vortex development of the shed TEV the after dorsal stroke reversal (t=0s) and at 9 equally-spaced times ranging from t=0.04s ($\hat{t}$=0.01, eq. 7) to t=0.36s ($\hat{t}$= 0.087, eq. 7) and at 9 spanwise, equally-spaced distances from the wing hinge to tip (Fig. S4). First, we estimated axial (horizontal) velocity along the TEV's core (Fig. S5a). At 0.6*R*, axial velocity is 0.032 ms^{-1} and equal to ~38% the vertical downwash velocity (Fig. S5b). Second, we scored sectional kinetic energy and total circulation of TEV and normalized these values to circulation at 0.6*R* (Fig. S6). Assuming that the circulation profile is displaced spanwise towards the wing tip at 0.032 ms^{-1} during a time (~0.42s) that corresponds to the fly's wing fling-peel time (t_{peel}=~1ms), TEV sectional energy change by not more than ~36.6% and circulation by ~21.93% of their initial values (t=0) at 0.6*R* (Fig. S6). These values are significantly smaller than the measured decrease in LEV strength and thus may not fully explain our measurements in the fly.

According to the available data, a shift in LEV that may fully explain the loss in LEV's sectional energy during fling-peel (~71% of initial value) would require a spanwise velocity of at least ~0.63 ms^{-1}. The latter value is similar to mean vertical down wash velocity (~0.64 ms^{-1}) measured in the *Drosophila* wake[17]. Our data provide no evidence that the fruit fly generates such strong axial flow components.

Supplementary text S5: Robotic validation - trailing edge vortex decay

To validate decay rate of shed TEVs in the fly (Fig. S3), we experimentally determined TEV vorticity dissipation in model wings at the beginning of the downstroke (Fig. S7). For comparison, we used normalized forms of circulation, sectional kinetic energy, and time in both experiments (i.e. fly and physical model) as suggested by Mohnseni and Gharib[18]. The non-dimensional values were calculated from,

$$\hat{\Gamma} = \frac{\Gamma}{c(c\omega_r + Ru_t)}, \quad \hat{E}_{SEC} = \frac{E_{SEC}}{\rho[c(c\omega_r + Ru_t)]^2}, \text{ and } \hat{t} = \frac{t}{T}, \qquad \text{(eq. S11)}$$

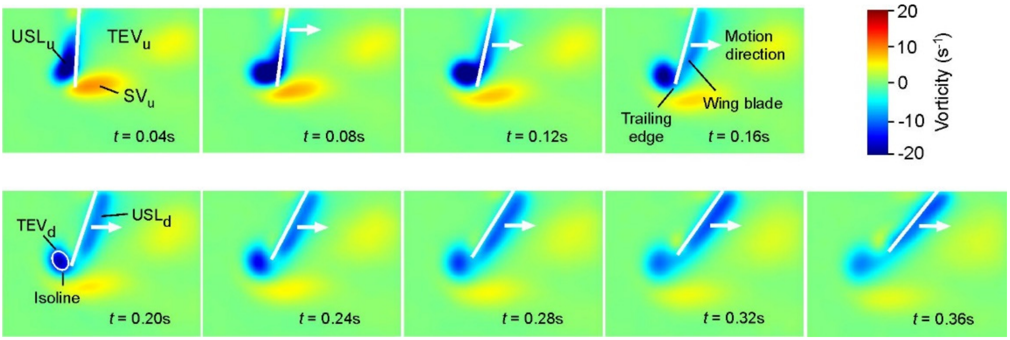

Fig. S7 | Vortex development of a trailing edge vortex shed at the beginning of a downstroke in a *Drosophila* model wing. Time series show wake development after the dorsal stroke reversal (t=0 ms). USL_u, undershear layer during upstroke; USL_d, undershear layer during downstroke; TEV_u, trailing edge vortex shed at the start of the upstroke; TEV_d, trailing edge vortex shed at the beginning of the downstroke;. SV_u, stop vortex at trailing edge at the end of the upstroke. See text for wing kinematics. Wing blade (white) is shown at 0.6 wing length, with the leading edge pointing upward.

with ω_r and u_t the wing's rotational and translational velocities, respectively, and T the period of the complete stroke cycle. Kinematic pattern and wing shape were similar to the experiments described in supplementary text S4. The temporal decay in non-dimensional circulation and sectional kinetic energy was eventually estimated employing a linear regression model.

The analysis shows that the slopes of the TEV's normalized sectional kinetic energy are –1.40 (left wing fruit fly, Fig. S3a), –0.72 (right wing fruit fly, Fig. S3a), and –0.86 (robotic wing). The slopes of normalized circulation are –2.24 (left wing fruit fly, Fig. S3b), –1.31 (right wing fruit fly, Fig. S3b), and –1.96 (robotic wing). The results suggest that freely moving TEVs dissipate at similar speed in both the fly and the physical model, which supports our conclusion that the measured decrease in kinetic energy of the fly's LEV requires an energy transfer to the nearby wing surface.

Supplementary text S6: Impact of vortex stretching on dissipation

Theory

This section evaluates the significance of vortex stretching on vortex dissipation in the fruit fly. In a viscous fluid, the dissipation rate ε is given via the enstrophy, $\varepsilon = 2\nu Z$, which is $Z = \int \vec{\omega} \cdot \vec{\omega}\, d\Omega$. To explain the time evolution of enstrophy and, equivalently, dissipation rate, we use the classical derivation. Our starting point is the incompressible Navier–Stokes equation written with primitive (u, p) variables,

$$\begin{aligned} \partial_t \vec{u} + (\vec{u} \cdot \nabla)\vec{u} &= -\nabla p + \nu \nabla^2 \vec{u} \\ 0 &= \nabla \cdot \vec{u}. \end{aligned} \qquad \text{(eq. S12)}$$

We first apply the curl to the preceding equations that yields the vorticity equation,

$$\underbrace{\partial_t \vec{\omega} + \vec{u} \cdot \nabla \vec{\omega}}_{\text{transport}} = \underbrace{\nu \nabla^2 \vec{\omega}}_{\text{dissipation}} + \underbrace{\vec{\omega} \cdot \nabla \vec{u}}_{\text{vortex-stretching}} . \qquad \text{(eq. S13)}$$

The last term on the right hand side disappears in two-dimensional flows and is called vortex stretching. Both equations are only valid for periodic flows without boundary conditions and thus not directly applicable to our experiments with a moving fluid–solid interface (wing).

For the numerical simulation, we thus employed the volume penalization method that is well established among the immersed boundary methods[19,20]. It adds a supplementary term to the Navier–Stokes equation, i.e.,

$$\begin{aligned} \partial_t \vec{u} + (\vec{u} \cdot \nabla)\vec{u} &= -\nabla p + \nu \nabla^2 \vec{u} - \chi / C_\eta (\vec{u} - \vec{u}_s) \\ 0 &= \nabla \cdot \vec{u}, \end{aligned} \qquad \text{(eq. S14)}$$

with χ the indicator function ($\chi = 0$ in the fluid, $\chi = 1$ in the solid, $0 < \chi < 1$ in a thin layer near the interface[21]), $C_\eta \ll 1$ the penalization constant called 'permeability', and $\vec{u}_s$ the velocity of the solid body. Considering the fluid's curl, the penalized vorticity equation may be written as[21,22],

$$\underbrace{\partial_t \vec{\omega} + \vec{u} \cdot \nabla \vec{\omega}}_{\text{transport}} = \underbrace{\nu \nabla^2 \vec{\omega}}_{\text{dissipation}} + \underbrace{\vec{\omega} \cdot \nabla \vec{u}}_{\text{vortex-stretching}} - \underbrace{\nabla \times \frac{\chi}{C_\eta} (\vec{u} - \vec{u}_s)}_{\text{boundary/penalization}} . \qquad \text{(eq. S15)}$$

We dot-multiplied the latter equation with $\vec{\omega}$ to eventually obtain the penalized enstrophy equation,

$$\partial_t Z = \underbrace{-\vec{\omega}\cdot\vec{u}\cdot\nabla\vec{\omega}}_{\text{transport}} + \underbrace{\vec{\omega}\cdot\nu\nabla^2\vec{\omega}}_{\text{dissipation}} + \underbrace{\vec{\omega}\cdot\vec{\omega}\cdot\nabla\vec{u}}_{\text{vortex-stretching}} \underbrace{-\vec{\omega}\cdot\nabla\times\frac{\chi}{C_\eta}(\vec{u}-\vec{u}_s)}_{\text{boundary}}. \qquad \text{(eq. S16)}$$

The temporal evolution of enstrophy is governed by transport, dissipation, vortex stretching and the boundary term. In index notation, vortex-stretching reads[23],

$$\begin{aligned}\omega_i\omega_j\frac{\partial u_i}{\partial x_j} = \quad & \omega_x\omega_x\frac{\partial u_x}{\partial x} + \omega_y\omega_x\frac{\partial u_y}{\partial x} + \omega_z\omega_x\frac{\partial u_z}{\partial x} + \cdots \\ & \omega_y\omega_x\frac{\partial u_x}{\partial y} + \omega_y\omega_y\frac{\partial u_y}{\partial y} + \omega_z\omega_y\frac{\partial u_z}{\partial y} + \cdots \\ & \omega_z\omega_x\frac{\partial u_x}{\partial z} + \omega_z\omega_y\frac{\partial u_y}{\partial z} + \omega_z\omega_z\frac{\partial u_z}{\partial z}.\end{aligned} \qquad \text{(eq. S17)}$$

We may easily calculate vortex stretching because eq. S17 only contains first derivatives of velocity $\vec{u}$. Transport- and dissipation terms, by contrast, are more challenging to obtain because these terms contain higher order spatial derivatives of velocity (up to order of 3) that leads to elevated high-frequency errors. The latter holds, in particular, for flows near the boundary layer at which the solution is in C^1. In our simulation, we thus used the following definitions for vortex stretching and enstrophy dissipation,

$$\begin{aligned}\text{(vortex stretching)} &= \int_{\Omega_{\text{fluid}}} \vec{\omega}\cdot\vec{\omega}\cdot\nabla\vec{u}\,d\Omega \\ \text{(enstrophy dissipation)} &= \int_{\Omega_{\text{fluid}}} \vec{\omega}\cdot\nu\nabla^2\vec{\omega}\,d\Omega,\end{aligned} \qquad \text{(eqs. S18)}$$

without considering the solid domain and the regions near the fluid-solid interface. As $\chi \equiv 0$ in Ω_{fluid}, the boundary term is omitted.

Numerical results

We calculated vortex stretching using our numerical code (https://github.com/adaptive-cfd/WABBIT) for wavelet-adaptive simulation of insect flight (https://arxiv.org/abs/1912.05371). We approximated wing geometry and kinematics from previous studies but without considering prescribed chordwise wing deformation or full fluid–structure interaction coupling with deformable wings.

Wing kinematics was broadly adopted from a generalized pattern[20]. However, as this pattern does not include clap–fling kinematics, we also used data from a study[24] on wing motion in the small wasp *Encarsia formosa*. Wing kinematics in this animal is qualitatively similar to the kinematics of a fruit fly. We visualized the kinematic model in figure S8. Other parameters of the fruit fly model[25] are wing length $R = 2.37 \cdot 10^{-3}$ m, mean chord length $c_m = 2.94 \cdot 10^{-3}$ m, frequency $f = 210$ Hz, fluid density $\varrho = 1.246$ kg/m^3 and fluid viscosity $\nu = 15 \cdot 10^{-6}$ m^2/s. Using a stroke amplitude $\Phi = 142°$, conventional Reynolds number $Re = u_{tip}c_m/\nu$ equals 120.

In order to ensure reliable numerical results, we performed a set of three simulation runs using coarse, medium and fine resolutions of the underlying numerical grid. Our numerical code automatically adapts the grid refinement to the flow field that limits the maximum number of consecutive rounds of overall refinement. In the 'coarse simulation', we allow the code to refine the computational grid up to six times that yields a grid spacing Δx equal to $5.7 \cdot 10^{-3}R$ or 13 µm. This value for Δx represents the upper bound of resolution in the simulation and is used only where required. In the medium (fine) resolution case, we added one (two) grid refinements by a factor of two. Here, minimum grid spacing equals $2.8 \cdot 10^{-3}R$ ($1.4 \cdot 10^{-3}R$). For further details on the numerical method including the choice of parameters and validation tests, we refer to https://arxiv.org/abs/1912.05371 and previous results[20,21,26].

Figure S9 shows that fluid vorticities ($|\omega| = 50$) obtained from medium and fine resolution simulations are quite similar. Figure S10 shows the time evolution of kinetic energy E_{kin}, enstrophy Z and the time derivative of enstrophy dZ/dt as a function of time. For this convergence test, E_{kin} and Z are integrated over the entire domain Ω. E_{kin} is very similar among the three tested grid resolutions, while enstrophy slightly differs at mid up and mid downstroke. Figure S11 finally highlights the contributions of enstrophy dissipation and vortex stretching to the time evolution of enstrophy (eqs. S18). The latter data are computed with fine grid resolution, while computations with medium resolution are used for error estimation.

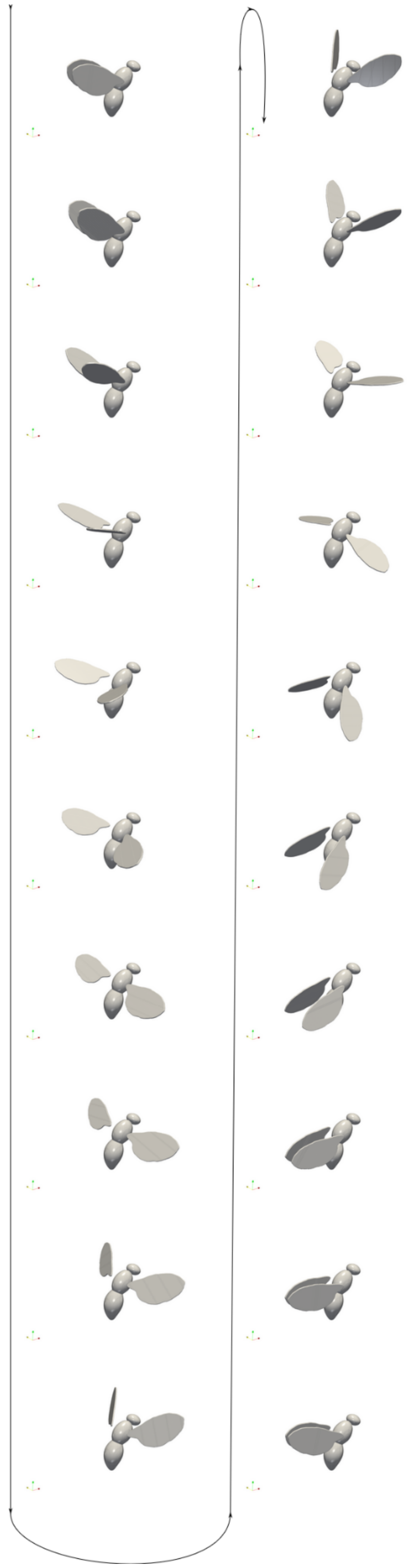

Fig. S8 | Wingbeat kinematics of the simulated fruit fly model for evaluation of vortex stretching. Figure shows wing positions at 20 equally-spaced times of the wing stroke cycle. Wing and body geometry including wing kinematics are adopted from previously published data[20,24,27].

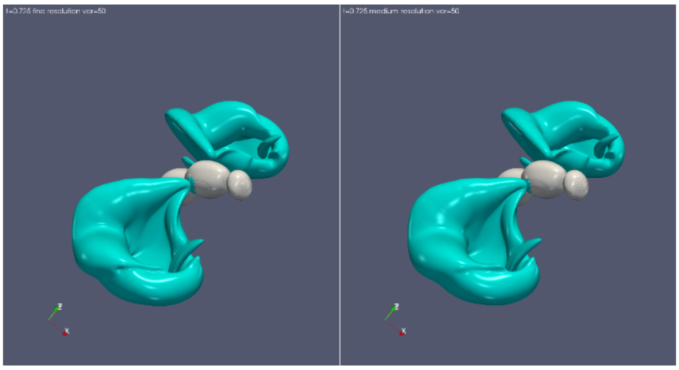

Fig. S9 | Comparison of flow fields at t/T=0.725 stroke cycle computed using medium (right) and fine (left) resolution simulation. Data show |ω|=50 isosurface of vorticity magnitude.

Time evolution of vortex stretching is shown in figure S11 (orange). Its error is comparatively small and thus rather independent from grid resolution (Fig. S11, shaded area). The uncertainty of enstrophy dissipation, by contrast, is significantly larger and monotonically increases with increasing grid resolution (coarse to fine). Thus, although enstrophy dissipation is likely somewhat underestimated, it is yet approximately one order of magnitude larger than vortex stretching. We conclude that vortex stretching is negligible compared to enstrophy dissipation at Reynolds numbers relevant to flight of fruit flies.

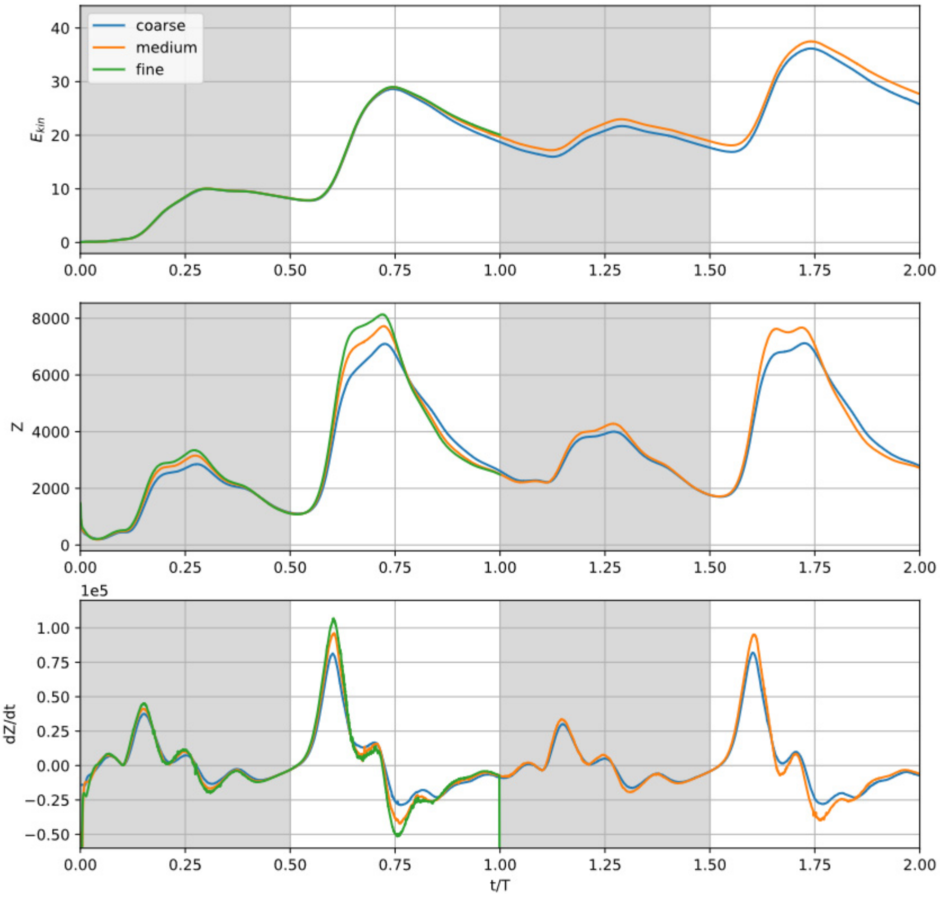

Fig. S10 | Parameters of simulation using coarse, medium and fine numerical resolutions. Data show kinetic energy E_{kin}, enstrophy Z and its time derivative dZ/dt as a function of time normalized by cycle duration T. Gray shaded area corresponds to the wings' downstroke. High resolution data stem from a single simulated flapping cycle. Integration is performed over the full domain Ω.

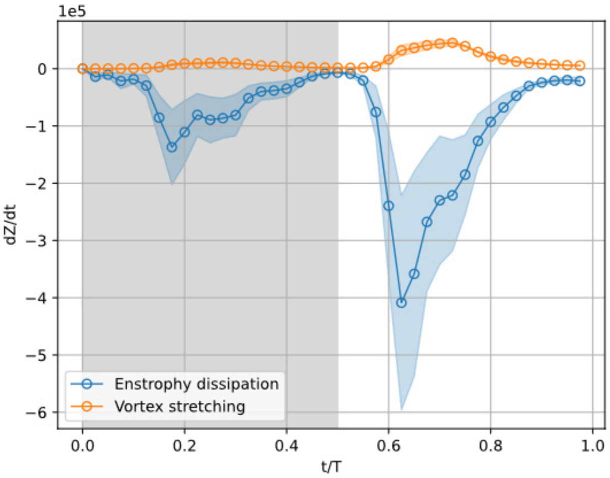

Fig. S11 | Contribution of enstrophy dissipation and vortex stretching (eqs. S18) to the time evolution of enstrophy (eq. S16). Integration was performed in Ω_{fluid}, excluding the regions in and near the solid wings and body. The shaded areas show the difference between numerical results obtained from medium and fine grid resolutions. Gray indicates the wings' downstroke during wing stroke cycle T.

References

1 Weis-Fogh, T. Quick estimates of flight fitness in hovering animals, including novel mechanisms for lift production. *J. Exp. Biol.* **59**, 169-230 (1973).

2 Lehmann, F. O., Sane, S. P. & Dickinson, M. H. The aerodynamic effects of wing-wing interaction in flapping insect wings. *J. Exp. Biol.* **208**, 3075-3092 (2005).

3 Bartussek, J. & Lehmann, F.-O. Proprioceptive feedback determines visuomotor gain in *Drosophila*. *R. Soc. Open Sci.* **3**, 150562, doi:10.1098/rsos.150562 (2016).

4 Shishkin, A., Schützner, P., Wagner, C. & Lehmann, F.-O. in *Nature-Inspired Fluid Mechanics* (eds C. Tropea & H. Bleckmann) 81-99 (Springer, 2012).

5 Marden, J. H. Maximum lift production during takeoff in flying animals. *J. Exp. Biol.* **130**, 235-258 (1987).

6 Marden, J. H. Effects of load-lifting constraints on the mating system of a dance fly. *Ecology* **70**, 496-502 (1989).

7 Jeong, J. & Hussain, F. On the identification of a vortex. *J. Fluid Mech.* **285**, 69-94 (1995).

8 Batchelor, G. K. *An introduction to fluid dynamics*. (Cambridge university press, 2000).

9 Saffman, P. G., Ablowitz, M. J., Hinch, J., Ockendon, J. R. & Olver, P. J. *Vortex dynamics*. 253 (Cambridge University Press, 1992).

10 Dzieddzic, M. & Leutheusser, H. J. An experimental study of viscous vortex rings. *Exp. Fluids* **21**, 315-324 (1996).

11 Maybury, W. J. & Lehmann, F.-O. The fluid dynamics of flight control by kinematic phase lag variation between two robotic insect wings. *J. Exp. Biol.* **207**, 4707-4726 (2004).

12 Clercx, H. J. H. & van Heist, G. J. F. Dissipation of kinetic energy in two-dimensional bounded flows. *Phys. Rev. E* **65**, doi:10.1103/PhysRevE.65.066305 (2002).

13 Lehmann, F.-O. & Dickinson, M. H. The changes in power requirements and muscle efficiency during elevated force production in the fruit fly, *Drosophila melanogaster. J. Exp. Biol.* **200**, 1133-1143 (1997).

14 Kolomenskiy, D. *et al.* Aerodynamic ground effect in fruitfly sized insect takeoff. *PLoS ONE* **11**, doi:10.1371/journal.pone.0152072 (2016).

15 Pick, S. & Lehmann, F.-O. Stereoscopic PIV on multiple color-coded light sheets and its application to axial flow in flapping robotic insect wings. *Experients in Fluids* **47**, 1009-1023 (2009).

16 Birch, J. M. & Dickinson, M. H. Spanwise flow and the attachment of the leading-edge vortex on insect wings. *Nature* **412**, 729-733 (2001).

17 Berthé, R. & Lehmann, F.-O. Body appendages fine-tune posture and moments in freely manoeuvring fruit flies. *J. Exp. Biol.* **218**, 3295-3307, doi:10.1242/jeb.122408 (2015).

18 Mohseni, K. & Gharib, M. A model for universal time scale of vortex ring formation. *Phys. Fluids* **10**, 2436-2438 (1998).

19 Angot, P, Bruneau, C. & Fabrie, P. A penalization method to take into account obstacles in incompressible viscous flows. *Numer. Math.* **81**, 497–520 (1999).

20 Engels, T., Kolomenskiy, D., Schneider, K. & Sesterhenn, J. FluSI: A novel parallel simulation tool for flapping insect flight using a Fourier method with volume penalization. *SIAM J. Sci. Comput.* **38**(5), S3–S24 (2016).
21 Engels, T., Kolomenskiy, D., Schneider, K. & Sesterhenn, J. Numerical simulation of fluid-structure interaction with the volume penalization method. *J. Comput. Phys*. **281**, 96–115 (2015).
22 Engels, T., Kolomenskiy, D., Schneider, K. & Sesterhenn, J. Two-dimensional simulation of the fluttering instability using a pseudospectral method with volume penalization. *Computers & Structures* **122**, 101–112 (2012).
23 Pope, S. B. Turbulent flows. Cambridge University Press 2001.
24 Cheng, X. & Sun, M. Very small insects use novel wing flapping and drag principle to generate the weight-supporting vertical force. *J. Fluid Mech.* **855,** 646–670 (2018).
25 Engels, T., Wehmann, H.-N. & Lehmann, F.-O. Three-dimensional wing structure attenuates aerodynamic effciency in flapping fly wings. *J. R. Soc. Interface* **17**, 20190804, http://dx.doi.org/10.1098/rsif.2019.0804.
26 Kolomenskiy, D., & Schneider, K. A Fourier spectral method for the Navier-Stokes equations with volume penalization for moving solid obstacles. *J. Comput. Phys.* **228**, 5687–5709 (2009).
27 Maeda, M., & Liu, H. Ground effect in fruit fly hovering: A three-dimensional computational study. *J. Biomech. Sc. Engin.*, **8**, 344–355 (2013).

Caption Movie S1

The movie shows flow conditions during the dorsal stroke reversal in a tethered flying fruit fly *Drosophila virilis*. The first part of the movie shows motion of seeding particles in the horizontal laser sheet oriented normal to the wings' longitudinal axes. The second part shows vortex development and vorticity distribution as calculated from particle image velocimetry. Video recording rate is 10 kHz and wing beat frequency ~150 Hz.
